# Evolution of transfer RNA and the origin of the translation system

**DOI:** 10.3389/fgene.2014.00303

**Published:** 2014-08-28

**Authors:** Savio T. de Farias, Thaís G. do Rêgo, Marco V. José

**Affiliations:** ^1^Laboratório de Genética Evolutiva Paulo Leminsk, Departamento de Biologia Molecular, Universidade Federal da ParaíbaJoão Pessoa, Brazil; ^2^Departamento de Informática, Universidade Federal da ParaíbaJoão Pessoa, Brazil; ^3^Theoretical Biology Group, Instituto de Investigaciones Biomédicas, Universidad Nacional Autónoma de MéxicoMéxico, México

**Keywords:** tRNA, aminoacyl-tRNA synthetases, co-evolution, translation system, origin of life

The origin of the translation system is at the center of discussions about the evolution of biological systems. In this context, molecules of transfer RNA (tRNA) are highlighted due to its ability to convey the information contained in nucleic acids with the functional information contained in the proteins. Despite many characteristics shared among tRNAs in various organisms, suggesting a monophyletic origin for this group of molecules, recent discussions have proposed a polyphyletic origin for this group, thus indicating that the shared features are products of evolutionary convergence (Di Giulio, 2013). The main arguments in favor of the model for polyphyletic origin of tRNAs, is based on the theory of exons and suggests that the introns played an important role uniting mini exons or genes, which enabled that minigenes with independent origins were grouped in a single transcription unit at the start of the biological system (Di Giulio, 2012a). Genes for tRNAs have one of the most conserved introns that we know, which would represent a remnant of the process that gave rise to this molecule, being the anticodon loop initially a minigene that was attached to the other loop or hairpin that gave origin to the modern structure of tRNAs (Di Giulio, 2012b). An evidence of this model was found in *Nanoarchaeum equitans*, where a single tRNA is encoded by two genes that are united after the transcription (Randau et al., 2005). Podar et al. (2013), analyzed the genome of *N. equitans* and suggested that the organization of genes in this organism is a derived character, being consequence of a process of genomic reduction that is associated with their lifestyle (Podar et al., 2013). Thus, the tRNAs would be monophyletic, having a single ancestor that gave origin to the diversity known today, as suggested by Lacey and Staves (1990).

## Phylogenetic relationships among the tRNAs and co-evolution with the aminoacyl tRNA synthetases

**Figure 1:**
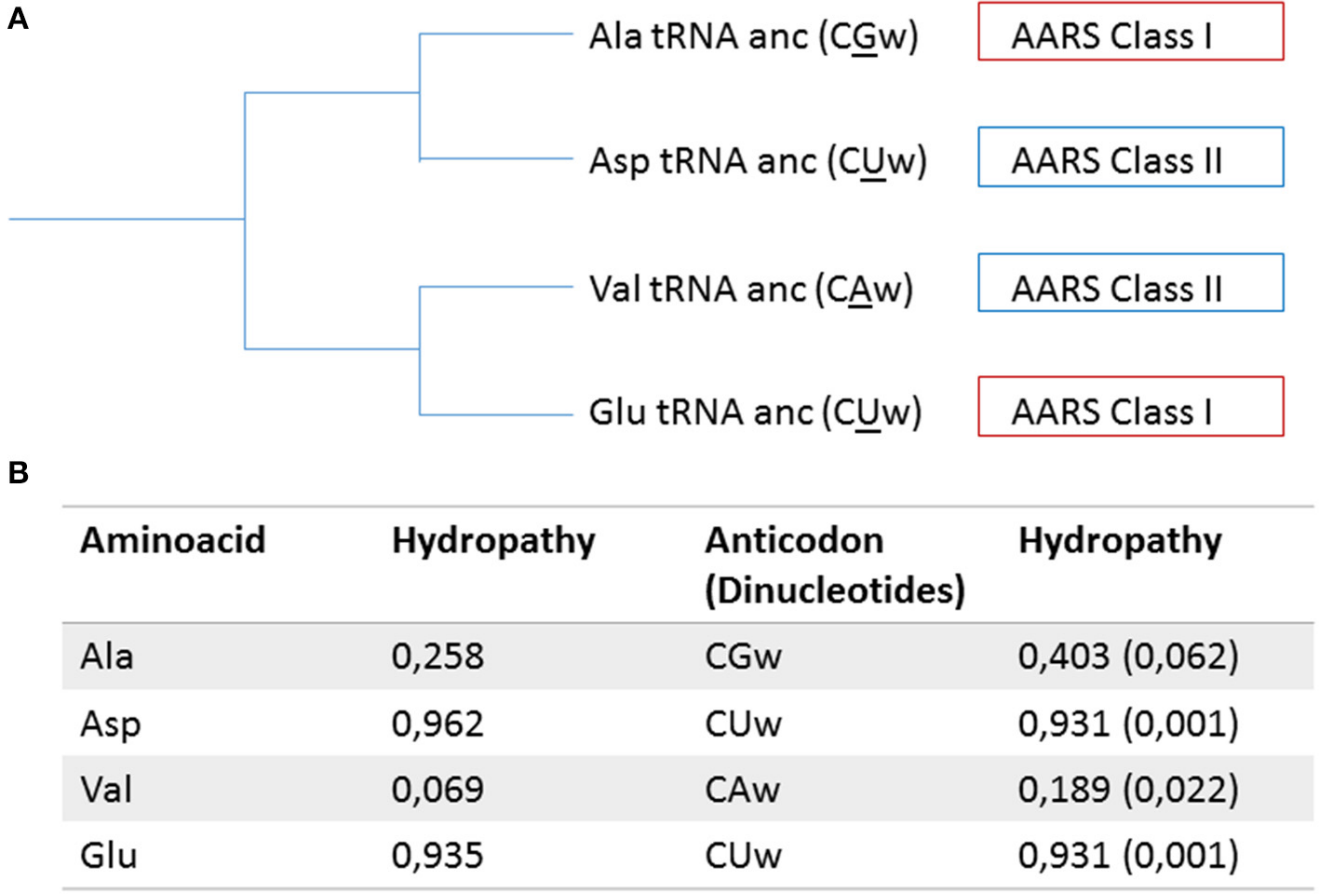
**Model for the selection mechanism between tRNAs and aminoacyl-tRNA synthetases**. In **(A)** A branch of the tRNA phylogeny (derived from Farias et al., 2014), where the anticodons are in parentheses, w corresponds to the wobble position and the second base of the anticodons are underlined; the corresponding class of each aminoacyl-tRNA synthetase is indicated in the boxes. **(B)** The hydropathy of aminoacids and their corresponding anticodons (adapted from Farias et al., 2007). The pattern of diversification of tRNAs, via modifications of the second base of the anticodon, led to changes in hydropathy of the anticodon, and was accompanied by changes in the class of aminoacyl-tRNA synthetases responsible for the recognition and assignment of a new amino acid.

Lacey and Staves (1990) proposed that the tRNA molecules have a monophyletic origin, with modern molecules derived from the universal translator. The conservation of the structure, sequences of certain regions in tRNA, and the position of the introns, reinforce the monophyletic origin of this class of molecules. Farias (2013) by assuming the monophyly of tRNA molecules, proposed a new model for the diversification of these molecules. This study was based on the reconstruction of ancestral sequences of 22 types of tRNAs (20 canonical, 1 initiator and 1 selenocysteine) and he suggested that the driving force in the diversification process was due to changes in the second base of the anticodon, a pattern also observed in Wang and Lavrov (2011). Hydropathy analysis of the pattern of amino acids and anticodons, show a direct correlation between the type of amino acid and their respective anticodon. This could indicate an event of the direct co-evolution in the process of diversification of tRNAs and in the entry of new amino acids in protein composition. However, from the analysis of the organization of the modern genetic code, we can see that changes in the second base of the anticodon change the hydropathy of anticodons and in most cases leads to a change of the class of aminoacyl-tRNA synthetase responsible for recognizing specific tRNA (Farias et al., 2007). Studies conducted to reconstruct the phylogenetic relationships of aminoacyl-tRNA synthetases show a pattern of diversification associated with characteristic hydropathy of the specific amino acid for each type of enzyme (Nagel and Doolittle, 1995; Farias and Guimarães, 2008). The data obtained for the evolutionary history of tRNA and aminoacyl-tRNA synthetases suggest that the hydropathy correlation observed between amino acids and their anticodon may have occurred indirectly via affinity of catalytic sites of aminoacyl tRNA synthetase by a specific type of hydropathy similar to the one observed between the anticodon and amino acid and not by a direct diversification process, as suggested in Figure 1. In this context, the pattern of diversification of tRNAs could have been selected to minimize binding of tRNAs from the same ancestry with aminoacyl-tRNA synthetases with the same pattern recognition, prompting the coevolution process between these two molecules having such a selective force to distinguish extreme hydropathy, which allowed for a primitive system and low specificity remained effectively. The process of diversification of tRNAs by changing the second base and consequently by changing hydropathy of the anticodon, may have been one of the forces for symmetric diversification between aminoacyl-tRNA synthetases. These data are consistent with the model proposed by Ribas de Pouplana and Schimmel (2001), who observed that the aminoacyl-tRNA synthetases Class I bind acceptor stem from minor groove side, while the aminoacyl-tRNA synthetases Class II bind to the major groove side of the acceptor stem. This data showed that the evolution of these molecules occurred symmetrically and was proposed that the recognition of tRNA by aminoacyl-tRNA synthetases occurred in a coordinated manner between the proteins of Class I and Class II. In this context, the anticodon loop must have originated in ancestral tRNAs and thus guided the establishment of the attribution currently observed between anticodons and amino acids, by indirect co-evolution with the aminoacyl-tRNA synthetases. Discussions on the initial emergence of the acceptor arm or anticodon arm remain open. Studies using micro tRNA containing the anticodon loop plus terminal CCA, demonstrated the ability to bond with amino acids, suggesting that the anticodon loop may have arisen early in the evolution of the tRNA molecules (Szathmáry, 1999). However, Sun and Caetano-Anollés (2008) proposed from structural analysis that the acceptor arm could have arisen first and the anticodon loop is a later event in the evolutionary history of tRNAs. Another evidence for this proposition was showed by structural analysis in aminoacyl-tRNA synthetases, where it is suggested that the anticodon recognition domain is a secondary event, being present in the ancestral molecule only the recognition site of the acceptor arm (Caetano-Anollés et al., 2013). The data for stereochemical correlation between anticodons and amino acids, as well as the data to diversification pattern of the tRNAs, suggest the importance of information contained in the anticodons for establishing correlations with the amino acids. In this way, if the acceptor loop was the primordial structure of tRNAs, this information appears currently contained in the anticodon loop and the acceptor loop may have had, in the early stages, an importance as a bifunctional adapter (Rodin et al., 1996).

## The role of tRNAs in the origin of ribosomes

The origin of ribosomes must have occurred in the early biological evolution and developed for a long period until the structure currently found. Among the various molecules that are part of the ribosome, we can emphasize the 23S subunit, because, in the V region of the 23S ribosomal RNA it is found the site of Peptidyl Transferase Center (PTC), the portion responsible for the ribozyme activity performed by this molecule (Nissen et al., 2000). Studies indicate that the region V was the first to emerge and from this region the ribosome was formed (Davidovich et al., 2009; Fox, 2010). A catalytic portion of the PTC has a structure with organization of the type stem-elbow-stem and experiments have shown that small molecules with this type of organization can polymerize and perform similar function that were observed for the PTC (Davidovich et al., 2009). The same type of structural organization (stem-elbow-stem) is also observed in the structure of tRNAs, thus, Tamura (2011) by topological analysis, suggested that the catalytic site on the 23S rRNA has structural similarities to tRNA molecules and may have originated from the fusion of tRNA molecules. Farias et al. (2014) compared the similarity of the reconstructed ancestral sequences of tRNA with the PTC of *Thermus thermophilus*, and they obtained a sequence similarity of 50.53% when concatamers were built with the ancestors of tRNAs, to wit, ^Leu^tRNA-^Ser^tRNA-^His^tRNA-^Pro^tRNA-^Tyr^tRNA-^Phe^tRNA-^Gln^tRNA-^Gly^tRNA-^Lys^tRNA and compared them with the portion of the catalytic of PTC. In this study the information content between the concatamers of ancestral tRNAs and the catalytic regions of PTC of various organisms were also compared, and a positive correlation among all molecules was observed, demonstrating that, despite the long evolutionary time, this molecule has vestiges of its early origin. This conservation must be essential for the maintenance of structural form assumed by the catalytic site in all species. Previous studies performed by Bloch et al. (1984, 1989), suggested the involvement of tRNAs in the formation of ribosomes. These analyzes suggested that the portion 5S Ribosomal presents similarities to tRNAs, and could have given rise to this ribosomal moeity. Evidence of the origin of the ribosomal RNAs from tRNA opens a new field of research, which may indicate a common origin for RNA molecules involved in the translation process. In this case, the translation system can be the oldest in the biologic system and we can indicate under what type of selective pressure the biological system evolved. Another field of research that opens up new perspectives is directly related to the origin and evolution of the coding system (genetic code) which must have arisen at the dawn of the early forms of life.

## Conclusion

Molecules of tRNAs play an essential role in modern biological systems. According to the data discussed, these molecules have had this function since the beginning of the formation and organization of biological systems. The evolutionary pattern of diversification of tRNA may indicate how the chemical relationship between these molecules and aminoacyl-tRNA synthesases could have co-evolved to give origin to the system of correlation between anticodons and amino acids. This suggestion can assist in developing models for the structure of the genetic code. The similarity observed between the tRNA molecules and ribosomal RNAs, especially the PTC, suggest the importance of the tRNAs molecules in the assembly of the translation system, which certainly was a key event for the emergence of life on Earth.

### Conflict of interest statement

The authors declare that the research was conducted in the absence of any commercial or financial relationships that could be construed as a potential conflict of interest.
